# Heavy Metals in Suspended Particulate Matter of the Zhujiang River, Southwest China: Contents, Sources, and Health Risks

**DOI:** 10.3390/ijerph16101843

**Published:** 2019-05-24

**Authors:** Jie Zeng, Guilin Han, Qixin Wu, Yang Tang

**Affiliations:** 1 Institute of Earth Sciences, China University of Geosciences (Beijing), Beijing 100083, China; zengjie@cugb.edu.cn; 2 Key Laboratory of Karst Environment and Geohazard, Ministry of Land and Resources, Guizhou University, Guiyang 550025, China; qxwu@gzu.edu.cn; 3 State Key Laboratory of Environmental Geochemistry, Institute of Geochemistry, Chinese Academy of Sciences, Guiyang 550081, China; tangyang@mail.gyig.ac.cn

**Keywords:** heavy metals, suspended particulate matter, enrichment, health risk, Pearl River, Southwest China

## Abstract

To investigate the abundance, water/particle interaction behavior, sources, and potential risk of heavy metals in suspended particulate matter (SPM), a total of 22 SPM samples were collected from the Zhujiang River, Southwest China, in July 2014 (wet season). Nine heavy metal(loid)s (V, Cr, Mn, Ni, Cu, Zn, As, Cd and Pb) in SPM were detected. The results show that the selected heavy metal(loid)s in SPM appear in the following order: Mn (982.4 mg kg^−1^) > Zn (186.8 mg kg^−1^) > V (143.6 mg kg^−1^) > Cr (129.1 mg kg^−1^) > As (116.8 mg kg^−1^) > Cu (44.1 mg kg^−1^) > Ni (39.9 mg kg^−1^) > Pb (38.1 mg kg^−1^) > Cd (3.8 mg kg^−1^). Furthermore, both the enrichment factor (EF) and geo-accumulation index (I_geo_) indicate that SPM is extremely enriched in metal(loid)s of Cd and As, while SPM is slightly enriched, or not enriched, in other heavy metals. According to the toxic risk index (TRI) and hazard index (HI), arsenic accounts for the majority of the SPM toxicity (TRI = 8, 48.3 ± 10.4%) and causes the primary health risk (HI > 1), and the potential risks of V and Cr are also not negligible. By applying a correlation matrix and principal component analysis (PCA), three principal components (PC) were identified and accounted for 79.19% of the total variance. PC 1 (V, Cr, Mn, Ni, Cu, and Pb) is controlled by natural origins. PC 2 (As and Cd) is mainly contributed by anthropogenic origins in the basin. PC 3 (Zn) can be attributed to mixed sources of natural and anthropogenic origins. Moreover, all the partition coefficients (lgK_d_) exceeded 2.9 (arithmetical mean value order: Mn > Pb > Cd > V ≈ Cu > Cr ≈ Ni), indicating the powerful adsorptive ability of SPM for these heavy metal(loid)s during water/particle interaction.

## 1. Introduction

Heavy metals are one of the most significant pollutants in the environment, particularly in the aquatic environment, that may cause severe deterioration of water quality and do harm to living organisms due to their toxicity, persistence, non-biodegradability, and bio-accumulation [1,2,3]. Generally, heavy metals in the aquatic system can be divided into three phases: dissolved load, suspended particulate matter (SPM), and sedimentary [4,5,6,7,8]. Although the dissolved phase is believed to be more toxic to aquatic organisms and humans, its content is usually lower than the suspended particle phase [9]. Because of the high surface area and reactivity of suspended particulate matter, the dissolved heavy metals are also easily absorbed by SPM [8,10]. Therefore, more attention has been paid to heavy metals of the suspended particle phase than those of the dissolved phase. Furthermore, as the major carrier and pre-sink of heavy metals in the fluvial environment [11], SPM in the aquatic system is not a threat for humans as a rule, but the main carrier of land materials export to the ocean [11,12]. Previous studies suggested that the bed load at estuaries accounts for less than 10% of a river’s total solids transported to the ocean and is often less than 1%, while more than 90% of solids are transported in suspension [13,14]. In addition, heavy metals in river water tend to accumulate in SPM because of its direct interface with the water, and the deposition of metal-adsorbed SPM is the primary process for the accumulation of heavy metals in bed sediments [10]. Accordingly, the contaminated surface bed sediment or deposited SPM might become re-suspended because of water flow disturbance [8,10]. This is a crucial process for the ecological risks of heavy metals across the sediment–water interface. Therefore, numerous studies regarding heavy metals in SPM, their effects on the fluvial environment, and the fluxes to the ocean have been published in various countries [4,6,11,12,15,16,17,18,19,20], including China [8,10,14,21,22,23]. A case study in the multi-anthropogenic polluted river in the Gulf of Tunis revealed that heavy metal (Pb, Cu, Zn, and Fe) pollution was mainly localized to commercial activities and fishing [4]. The study on the Tigris River showed that the dissolved phase dominated the physical speciation of many metals (low partition coefficients), but the Al, Fe, Pb, Th, and Ti exhibited high particulate fractions due to the high SPM concentration [6]. Viers et al. [11] presented a new database on the chemical composition (including heavy metals) of suspended matter in global rivers, together with the associated heavy metal fluxes, and they also give a “snap-shot” of heavy metal fluxes for each continent in order to assess the influence of human activities on natural geochemical cycles of heavy metals in different environments. A two-year monitoring data of particulate metals (Cd, Cu, Ni, Zn, Fe, Pb, Cr, and Mn) in an estuarine environment displayed no seasonal variation or any relationship with the tide, and the large input of particulate metals was attributed as probably being due to the intensive agriculture within the drainage basin [15]. Matsunaga et al. [19] explored the temporal variations in metal enrichment in SPM during rainfall events in a rural stream. In the Lerma River, particulate Fe and Mn originated predominantly from natural sources, whereas Cu, Zn, Cr, and Pb in SPM originated mainly from anthropogenic sources [16].

In terms of catchment management, identifying the contaminated level and ecological risk of heavy metals is a prerequisite for pollution remediation. Many methods (such as enrichment factor, anthropogenic metal flux, bioavailable metal index and toxic risk index) have been widely developed to evaluate the contaminated levels, anthropogenic inputs, bioavailability and toxicity of heavy metals in SPM or sediment [24]. The Zhujiang River is the largest river flowing into the South China Sea [25]; it is the major water source for a local population of about 30 million and provides important supports for the socio-economic development of southern China [26]. Since the intense anthropogenic disturbance on the Zhujiang River, several studies have been performed to investigate the heavy metal composition of the water [21,27], sediment [24,28,29,30], and SPM [8,21] in different reaches. These studies distinguished the heavy metal temporal transport of SPM in the upper reaches of the Zhujiang River and explored the partition coefficients of heavy metals between water and SPM in the tributaries of the lower reaches of the Zhujiang River. However, these studies were insufficient for obtaining a clear idea of the general status of the abundance and sources of heavy metals in SPM, and the water/particle interaction behavior of heavy metals in the Zhujiang River from a whole basin perspective. In addition, the risk assessment mainly focused on the bioavailability and toxicity of heavy metals in SPM (the major carrier and pre-sink of heavy metals) [5,8,24,28,29,31], while the health risk of human exposure has rarely been systematically reported up to now.

A previous study investigated the dissolved heavy metals in river water of the entire riverine system of the Zhujiang River [32]. However, it was impossible to get adequate SPM in all of the sites, particularly during the dry season. In the present study, an investigation on nine heavy metal(loid)s (V, Cr, Mn, Ni, Cu, Zn, As, Cd, and Pb) in 22 SPM samples in the Zhujiang River was conducted. The aims of this study were: (i) to analyze the enrichment of heavy metal(loid)s in SPM; (ii) to explore the behaviors of heavy metal(loid)s during water/particle interaction of the entire basin; (iii) to identify the sources of heavy metal(loid)s in SPM; and (iv) to assess the potential risk of heavy metal(loid)s in SPM, particularly to evaluate the health risk of human exposure firstly by referring to the U.S. EPA (Environmental Protection Agency) method. The results can be applied to increase prevention–control efficiency of heavy metal(loid) pollution as well as to prevent hazardous heavy metal(loid) pollution affecting the local people in the whole basin.

## 2. Materials and Methods

### 2.1. Study Area

**Figure 1:**
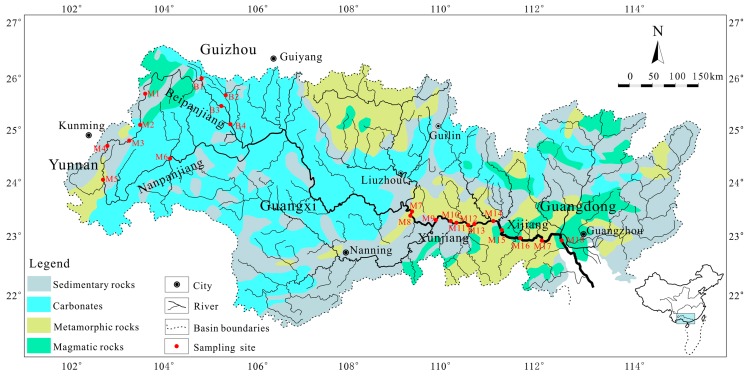
Map showing sampling locations and sample numbers of the Zhujiang River.

The Zhujiang River (21°31′–26°49′ N, 102°14′–115°53′ E) is the largest river flowing into the South China Sea and is the major water source for the population of more than 30 million in southern China [26,27]. As the elevation decreases from northwest to southeast, the Zhujiang River flows through Yunnan, Guizhou, Guangxi and Guangdong provinces with a coverage area of 4.5 × 10^5^ km^2^ (Figure 1). The Zhujiang River basin is characterized by a tropical to subtropical monsoon climate, where the annual temperature and annual precipitation range from 14 to 22 °C and 1200 to 2200 mm [25]. Various rocks, including carbonate rocks, metamorphic rocks, detrital sedimentary rocks, and magmatic rocks, are widely distributed in the Zhujiang River basin [25,26] (Figure 1). There are 24 large dams and 212 medium reservoirs located in the mid-lower reaches of the Zhujiang River [25].

### 2.2. Sample Collection and Analysis

Based on the lithology distribution, population distribution and reservoir/dam distribution of the Zhujiang River basin, 22 sampling sites were selected (Figure 1). Ten sites were located at the Nanpanjiang River (NPR, M1 to M6) and Beipanjiang River (BPR, B1 to B4) in the upper reaches of the Zhujiang River with widely distributed carbonates and a small population. Twelve sites were located at Xunjiang (XUR, M7 to M13) and Xijiang (XJR, M14 to M18) in the mid-lower reaches of the Zhujiang River, where there are large populations and many reservoirs/dams with metamorphic rock and magmatic rock development. Accordingly, a total of 22 river SPM samples were collected from the selected sites during July 2014 (wet season). The SPM samples in river water were firstly filtered through millipore nitrocellulose membrane filters, and the SPMs on the filter membranes were then removed by milli-Q water and dried at 55 °C in the laboratory. The digestion method of SPMs was modified from previous studies [21,33]. Briefly, 100 mg of SPM sample powder was digested with 1 mL pure HF and 3 ml pure HNO_3_ in a pre-cleaned PFA (Perfluoroalkoxy) sample jar (Savillex, Eden Prairie, MN, USA) at 140 °C. After the samples were completely digested, 2 mL pure HNO_3_ was added twice to break up residual fluorine compounds until evaporation to dryness. Finally, the remaining digest was re-dissolved using 100 mL 3% HNO_3_ for the heavy metal(loid) analyses. The heavy metal(loid)s (V, Cr, Mn, Ni, Cu, Zn, As, Cd, and Pb) of the digested solutions were determined by ICP-MS (Elan DRC-e, Perkin Elmer, Waltham, Massachusetts, USA), and the aluminum for the enrichment factor calculation was also detected by ICP-OES (Optima 5300DV, Perkin Elmer, Waltham, Massachusetts, USA). All the samples and standards were analyzed in batches with a procedural blank. Relative standard deviations (RSD) for heavy metal(loid)s were ±5%.

### 2.3. Assessment Method and Statistical Analysis

#### 2.3.1. Enrichment Factor (EF)

The EF normalizes the content of a heavy metal(loid)s to a conservative element, and has been extensively used to assess the enrichment of heavy metal(loid)s quantitatively [20,24,33,34]. Here, Al was approved as a reference element due to its extensive distribution in continental rocks and scarcity in various pollution sources [35], and can be used to calculate the EF as follows [20,24]:

(1) $$\mathrm{EF}=[(C_{i}/C_{\mathrm{ref}}{)}_{\mathrm{SPM}}]/[(C_{i}/C_{\mathrm{ref}}{)}_{\mathrm{background}}]$$

where C_i_ is the concentration of the heavy metal(loid)s (mg kg^−1^), and C_ref_ is the concentration of the reference heavy metal(loid)s (mg kg^−1^). The (C_i_/C_ref_) ratio is calculated based on the local soil background values. The soil background values of the Yunnan and Guizhou provinces were used for NPR (M1 to M6), and BPR (B1 to B4) river reach samples, and the mean soil background values of Guangdong and Guangxi provinces were used for the downstream samples (M7 to M18) [36]. The corresponding enrichment level categorizations of the EF value [24] are listed in Table 1.

#### 2.3.2. Geo-Accumulation Index (I_geo_)

The geo-accumulation index (I_geo_) is also applied to assess the heavy metal(loid) contamination in SPM. This approach has been widely used in previous studies [8,20,37]. The I_geo_ is calculated as follows [38,39]:

I_geo_ = log _2_[C_i_/(1.5 × B_i_)] (2)

where C_i_ is the concentration of heavy metal i in the SPM (mg kg^−1^), B_i_ is the local soil background concentration of metal i (mg kg^−1^), and the coefficient 1.5 in the equation is used to minimize the effect of possible variations in the background values. The I_geo_ for each metal is classified using seven (0–6 grades) enrichment classes [38] (Table 1).

#### 2.3.3. Risk Assessment

The toxic risk index (TRI) is applied to assess the integrated toxic risk (mainly the potential ecological risk to aquatic organisms) based on both the threshold effect level (TEL) and the probable effect level (PEL) of heavy metal(loid)s. Here, we selected consensus-based TEL and PEL values [40], which have been successfully used to assess the potential ecological risks of aquatic system trace metal(loid)s in previous studies [5,24]. The TRI of the SPM is calculated by the following equation [5]:

(3) $$\mathrm{TRI}=\Sigma_{i=1}^{n}{\mathrm{TRI}}_{i}=\{[(C_{S}^{i}/C_{\mathrm{TEL}}^{i}{)}^{2}+(C_{S}^{i}/C_{\mathrm{PEL}}^{i}{)}^{2}]/2{\}}^{1/2}$$

where $C_{S}^{i}$ is the concentration of metal i (mg kg^−1^) in the SPM, $C_{\mathrm{TEL}}^{i}$ and $C_{\mathrm{PEL}}^{i}$ are the TEL and PEL of metal i (mg kg^−1^), respectively. The toxic risks are classified into five categories (Table 1) based on the TRI calculation [5].

The health risk of human exposure to SPM of the Zhujiang River was evaluated by referring to the U.S. EPA method [41], which considers the amount of metal(loid)s entering the body and the relationship between the undesirable health effects and reference dose. The non-carcinogenic risk is calculated and assessed by the hazard quotient (HQ) and hazard index (HI, the potential hazard to the human health). In general, direct ingestion and dermal absorption are the two main exposure pathways to heavy metal(loid)s in the aquatic system for human beings [42,43]. Since humans rarely drink water with SPM directly (direct ingestion), here we considered that dermal absorption is the only exposure pathway for heavy metal(loid)s in the SPM. The HQ is the ratio between exposure via each pathway and the reference dose (RfD). HI is the sum of the HQs for each heavy metal from all the pathways (in this study, HI is equal to HQ because there is only one pathway). If the HQ or HI exceeds 1, non-carcinogenic risk effects on human health are a concern, and further study is necessary. In contrast, there are no deleterious effects when HQ or HI is less than 1 [37,43]. The HQ and HI are calculated as follows [37,44]:

ADD_dermal_ = (C × EF × ED × SA × AF × ABS × 10^−6^)/(BW × AT) (4)

HQ = ADD/RfD(5)

HI = ΣHQs(6)

where ADD_dermal_ is the average daily doses by dermal absorption (mg kg^−1^ day^−1^); RfD is the reference dose (mg kg^−1^ day^−1^) [37,45], and the other parameters and values in these Equations (4)–(6) are shown in Table 2.

#### 2.3.4. Multivariate Analysis

Statistical approaches, including a correlation matrix and principal component analysis (PCA), were applied to analyze the dataset to obtain descriptive statistics and to explore the possible sources of the heavy metal(loid)s. PCA is the most common multivariate statistical method used to explore the associations and origins of heavy metal(loid)s [46], which could reduce the dimensionality of the dataset to several influencing factors while trying to preserve the relationships presented in the original data [43,47]. The factor contribution or variables with minor significance attained from PCA are further reduced by the varimax rotation method [43]. The results of PCA, including the factor loadings, eigenvalues, variance, and communalities, constitute the component matrix. The result of PCA is acceptable if the communalities value is close to 1. The factor loadings (the correlation coefficients between each principal component and initial variable) are classified as “strong”, “moderate”, and “weak” according to the absolute loading values of >0.75, 0.75–0.50, and 0.50–0.30, respectively [48]. In this study, PCA is performed for heavy metal(loid)s of SPM in the Zhujiang River to distinguish their possible origins. The suitability of the dataset for PCA was checked using the Kaiser-Meyer-Olkin (KMO) test and Bartlett’s sphericity test (*p* < 0.001) [47]. To avoid the numerical ranges of the original variables, the dataset was first standardized by a z-scale transformation.

### 2.4. Data Processing Method

For the statistical analyses of obtained data, Pearson’s correlation coefficient and principal component analysis (PCA) were performed using SPSS 21.0 (IBM, Armonk, NY, USA). The data were graphed with Origin 8.1 (EA, Redwood City, CA, USA) and Microsoft Office 2010 (Microsoft, Redmond, WA, USA) for Windows.

## 3. Results and Discussion

### 3.1. Abundance of Heavy Metal(loid)s in SPM

The concentrations of heavy metal(loid)s in SPM of the Zhujiang River are shown in Table 3. The Kolmogorov–Smirnov (K–S) test, which is a non-parametric test, was used to test the normal distribution of our data. The test results show that all parameters are normally distributed in the Zhujiang River (K–S test significance >0.1), and the arithmetical mean values of all parameters are suitable for comparison [43]. Therefore, the nine selected heavy metal(loid)s in this study can be ranked by abundance as follows: Mn (982.4 mg kg^−1^) > Zn (186.8 mg kg^−1^) > V (143.6 mg kg^−1^) > Cr (129.1 mg kg^−1^) > As (116.8 mg kg^−1^) > Cu (44.1 mg kg^−1^) > Ni (39.9 mg kg^−1^) > Pb (38.1 mg kg^−1^) > Cd (3.8 mg kg^−1^). Mn and Zn are the most abundant metals, with maximums of 1487.1 and 732.8 mg kg^−1^, respectively, compared to the soil background values of the Zhujiang River basin [36]. The concentrations of five metal(loid)s, including Cr, Mn, Zn, As, and Cd, in SPM are much higher than all soil background values, while the contents of the remaining metals are between the soil background values of several provinces. Cd concentration is 5.8–23.7 times higher than the soil background concentration values of the whole basin which can be considered the strongest enriched metal in SPM relative to the soil. Cr, Mn, Zn, and As concentrations are elevated 1.2–7.9 times the soil background concentration values.

On a global scale (Table 4), V, Cr, and Zn are generally close to the world average, Mn, Ni, Cu, and Pb are lower than the world average, while As and Cd are much higher than the world average [11]. Compared with the rivers in Asia (China), the contents of V, Cr, and Mn in SPM of the Zhujiang River are similar, Ni, Cu, and Pb are slightly lower, while Zn is slightly higher. Additionally, the metals (Cr, Ni, Cu, Zn, and Pb) easily affected by human activities in SPM of the Zhujiang River are much lower than those in Europe (with many developed countries), which partly reflects the impact of economic development on heavy metal pollution in the fluvial environment.

### 3.2. Water/Particle Interaction and Contamination Assessment

#### 3.2.1. Water/Particle Interaction

The partition coefficient (K_d_) is the ratio of the element content in solid form (SPM in this study) to dissolved content in water (ppm/ppm) [21], which provides empirical information about the water/particle interaction for trace metals [8,49] and is usually expressed as lgK_d_. A high lgK_d_ value signifies a powerful affinity of the metals with SPM [15]. In combination with the dissolved heavy metal concentration in the same water samples of Zhujiang River reported in our early work [32], the lgK_d_ values of the seven metals are calculated and summarized in Table 5. The lgK_d_ values of V, Cr, Mn, Ni, Cu, Cd, and Pb ranged from 3.6 to 5.0, 3.3 to 4.5, 4.7 to 7.0, 3.7 to 4.5, 2.9 to 5.3, 4.6 to 5.5, and 5.4 to 6.2, respectively. All the lgK_d_ values exceeded 2.9, indicating the powerful adsorptive ability of heavy metals for the SPM. The mean partition coefficients of seven metals decreased in the order of Mn > Pb > Cd > V ≈ Cu > Cr ≈ Ni (Table 5); mainly controlled by the ionic radius and particle reactivity of these metals and the particle size of the SPM [8,23,49]. Compared to some rivers in the world, the lgK_d_ values of seven metals are within the range of world river values [6,8,50,51,52,53] (Table 5). The partition coefficients of Cr, Cu, and Cd are comparable to some rivers in China [52], particularly the Beijiang River [8], a significant tributary of the lower reaches of the Zhujiang River. However, the lgK_d_ values of Mn, Ni, and Pb are relatively higher than those of rivers in China [8,52]. It is noteworthy that all the mean lgK_d_ values (except Pb) in the present study are lower than the monthly mean values of the upper Zhujiang River [21], which indicates the possible seasonal variations in water/particle interaction.

#### 3.2.2. Enrichment Factor

**Figure 2:**
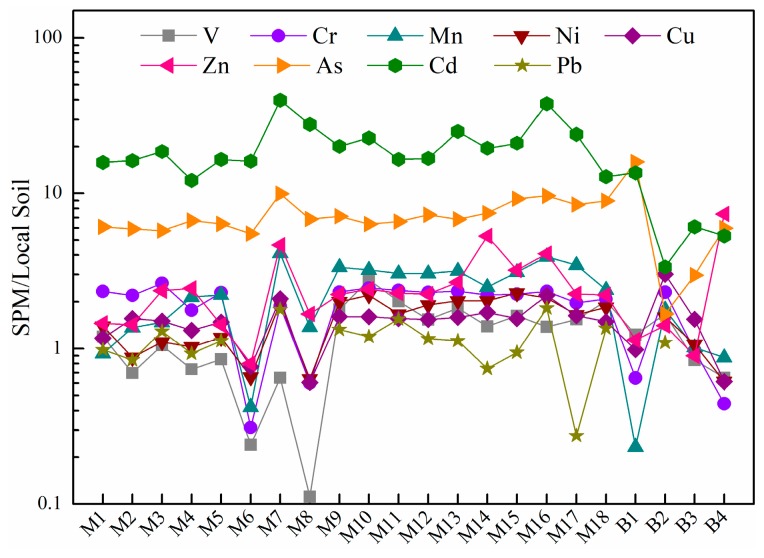
Abundances of nine heavy metal(loid)s in SPM normalized to local soil in the Zhujiang River.

The abundance of heavy metal(loid)s in SPM is normalized by the corresponding soil background values [36] in this study (Figure 2). Most metal(loid)s had a soil-normalized value which approached one and ranged from 0.1 to 4.1, with the exception of Zn, As and Cd. Soil-normalized values of As and Cd were 1.7 to 15.9 and 3.3 to 39.7, respectively, and indicate that all the SPM samples are enriched in metal(loid)s of As and Cd. Zn shows the soil-normalized value of varying degrees (0.8 to 7.4), which is more obvious in the headstream reach (M1 to M6, B1 to B4) and the XJR reach (M14 to M18).

**Figure 3:**
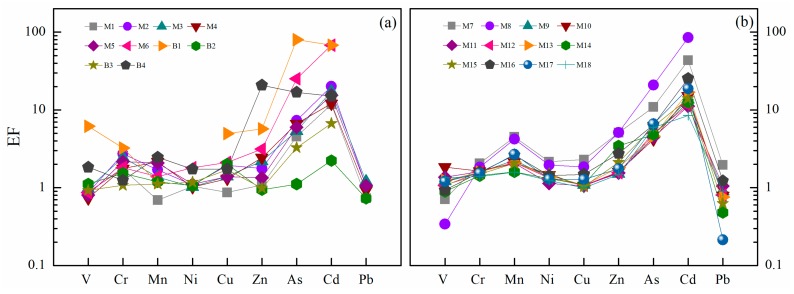
Enrichment factors (EF) of SPM in the Zhujiang River; (**a**) headstream, (**b**) downstream.

In order to quantitively evaluate the enrichment degree of heavy metal(loid)s in the Zhujiang River SPM, the enrichment factor (EF) was applied in the present study. As shown in Figure 3, the mean EF values of the SPM in all sites decreased in the order of Cd (23.3) > As (11.0) > Zn (3.2) > Mn (2.1) > Cr (1.8) > Cu (1.6) > Ni (1.4) > V (1.3) > Pb (0.9), indicating extremely severe enrichment of Cd and As. In the current study, the EF values of Cd in most sampling sites exceed 10 (severe enrichment, Table 1), and a few sampling sites exceed 50 (M6, M8, and B1), which can be defined as extremely severe enrichment (Table 1). The EF values of As mainly range from 5 to 10, which is a moderately severe enrichment. Cr, Mn, Ni, Cu, and Zn are slightly enriched, with mean EF values between 1.4 and 3.2, while the remaining metals (V and Pb) show no enrichment characteristics in most of the sites (EF < 1). It should be noted that the EF values of V (6.2), Cr (3.3), Cu (5.0), and As (79.8) are highest at B1, and the rest of the metals also have higher EF values, which illustrates that site B1 is the most strongly related to human activities [24]. Compared with the monthly SPM sampling of BPR (the mean EF values are 2.8, 3.1, 1.9, 2.7, 1.8, 2.4, 11.9, and 2.0 for V, Cr, Mn, Ni, Cu, Zn, Cd, and Pb, respectively) [21], most of the metals in our study have a relatively lower EF value, indicating that although the lgK_d_ values in this study (wet season) reflect the powerful adsorption capacity of SPM for heavy metals, there may be stronger water/particle interaction at the monthly scale, particularly particle adsorption. Furthermore, compared with the mean EF values of 11.0, 12.5, 10.0, 5.0, 19.6, and 19.6 for Cr, Ni, Cu, Zn, Cd, and Pb, respectively, in the polluted river (Soan River, Pakistan) [20], the enrichment degree of heavy metals in the Zhujiang River SPM is relatively slight.

#### 3.2.3. Geo-Accumulation Index

**Figure 4:**
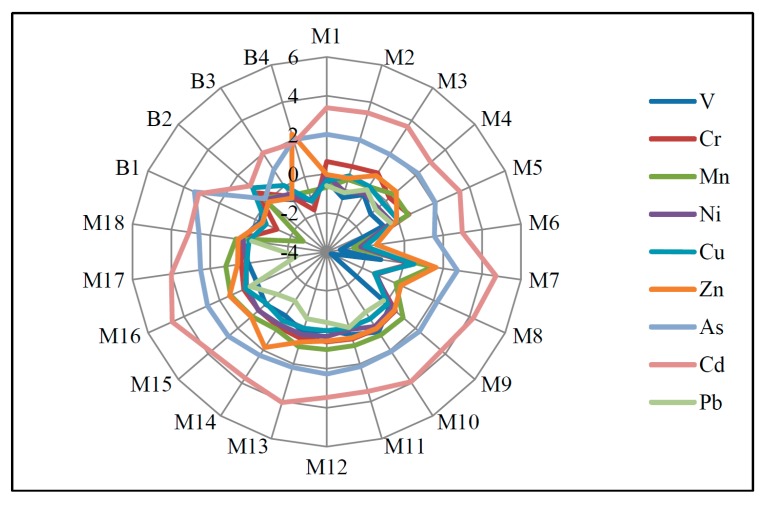
Geo-accumulation index (I_geo_) of heavy metal(loid)s of the SPM.

Based on the local soil background values (Table 3), the contamination degrees of heavy metal(loid)s in SPM of the Zhujiang River are assessed by the geo-accumulation index method (Equation (2)). The mean value of I_geo_ shows a contamination level order similar to EF (Cd > As > Zn > Mn > Cr > Cu ≈ Ni > V ≈ Pb, Figure 4). The most contaminated heavy metal(loid)s are Cd and As, with mean I_geo_ values of 3.4 and 2.1, respectively (Figure 4), revealing heavily polluted and moderately to heavily polluted levels. The mean value of I_geo_ for Zn (0.5), Mn (0.3), and Cr (0.1) classifies these metals as lightly polluted. The remaining metals (Cu, Ni, V, and Pb) have mean I_geo_ values of less than 0, indicating the unpolluted level (Figure 4). The mean I_geo_ values of the present study are consistently lower than those of the Beijiang River, an important tributary of the lower Zhujiang River, with several polymetallic mines and metal smelting enterprises (the mean values of I_geo_ are 2.1, 2.7, 3.1, 7.0, and 1.5 for Cu, Zn, As, Cd, and Pb, respectively) [8], revealing that the pollution intensity of heavy metal(loid)s in SPM is assuaged by the varying landscape setting of the whole Zhujiang River basin. This could be further confirmed by the comparison with polluted rivers [20].

### 3.3. Origins of Heavy Metal(loid)s in the SPM

#### 3.3.1. Correlation Analysis

A Pearson correlation matrix was employed to distinguish correlations between the nine heavy metal(loid)s of the SPM in the Zhujiang River (Table S1). The heavy metals with high correlation coefficients in the aquatic system could have similar sources, migration processes and chemical behavior [43,54]. In the current study, Cr, Mn, Ni, Cu, and Pb are remarkably positively correlated with each other (*p* < 0.01), indicating that these metals may be derived from the same source. Strong positive correlations are also observed between As and Cd (0.780), but these are poorly correlated with the remaining metals, suggesting that the sources of As and Cd are different from those metals. V is only significantly correlated with Cr (0.741), while Zn is not correlated with any metal (Table S1).

#### 3.3.2. Principal Component Analysis

**Figure 5:**
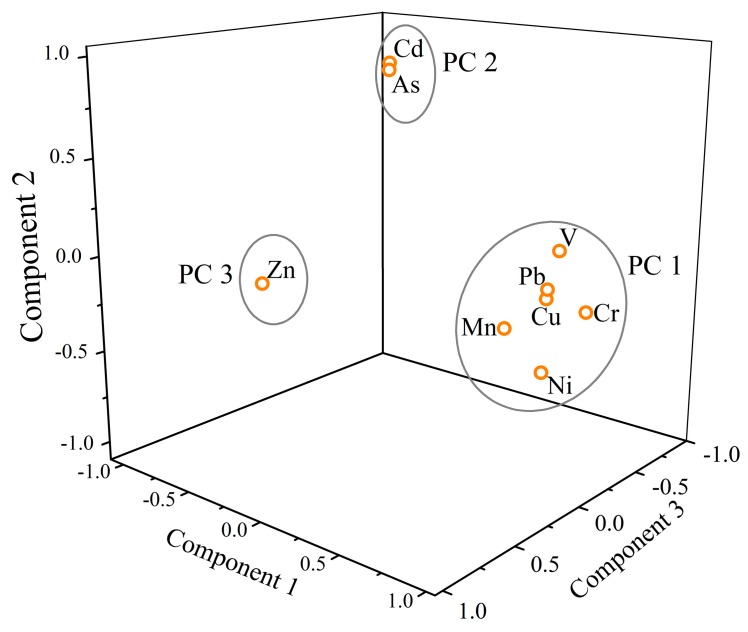
3D plot of scores for heavy metal(loid)s obtained from PCA results of SPM in the Zhujiang River.

In this study, PCA with the varimax rotation method was performed for heavy metal(loid)s of SPM in the Zhujiang River. There are three principal components (PC, eigenvalues >1) that are extracted and summarized in Table 6. PC 1 explains 44.51% of the total variance and predominantly includes V, Cr, Mn, Ni, Cu, and Pb. PC 2 explains 22.36% of the total variance with significant loadings of As and Cd. PC 3 explains 12.33% of the variance which is only contributed by Zn, and most of the heavy metal(loid)s exhibit a strong loading in their PCs (loading values >0.75) [48,55]. In total, these three PCs account for 79.19% of the total variance and are presented in a three-dimensional space, as shown in Figure 5. For PC 1, V is from lithophile elements [56], and Mn, Ni, and Cr are from natural sources of rock weathering and subsequent pedogenesis [24,57]. Although urban and industrial activities such as mining, metal smelting, and automobile exhausts can be the primary source of Cu and Pb [58], the lower EF values of Cu (1.6) and Pb (0.9) (Figure 3) indicate that the contribution of anthropogenic sources is limited [7,20]; hence, we attribute PC 1 to the natural origins controlled by geology and lithology. There are two metal(loid)s (As and Cd) with positive loadings on PC 2, and the correlation analysis suggests that the sources of As and Cd are different from those metals in PC 1. Considering the extremely high EF values of As (11.0) and Cd (23.3), we conclude that PC 2 is mainly contributed by anthropogenic origins in the basin [20,59]. In addition, Zn is the sole contributor to PC 3 and is not correlated with any metal (Table S1). In combination with the moderate enrichment of Zn (EF = 3.2), PC 3 can be attributed to mixed sources of geologic and anthropogenic origins.

### 3.4. Potential Risk Assessment and Heavy Metal(loid) Export Budget

#### 3.4.1. Toxic Risk Index (TRI)

**Figure 6:**
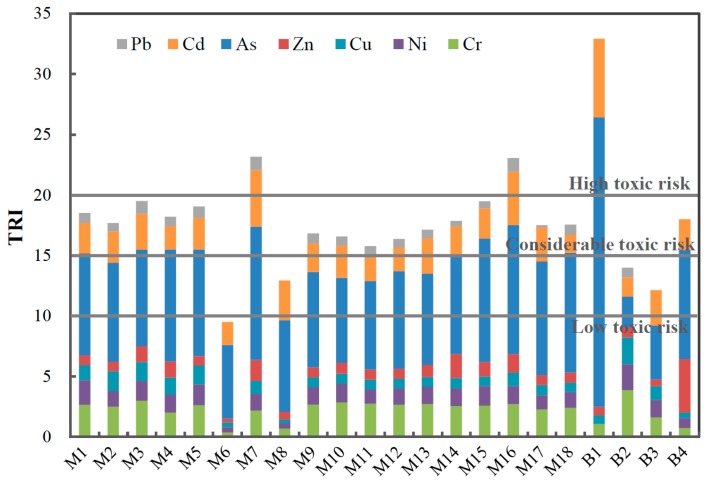
The toxic risk index (TRI) of heavy metal(loid)s of SPM in the Zhujiang River.

According to MacDonald [40], when the negative effects are less than 10% within the minimal effect range, the TEL is considered reliable, while the PEL is considered reliable if the negative effects exceed 65% of the probable effect range [5,40]. Thus, the TRI integrating the TEL and PEL, does not consider only the acute toxicity but also the lasting chronic toxic effects of heavy metals [24]. Based on the consensus TEL and PEL values [40] in (Table 3) and Equation (3), the TRI of seven metal(loid)s were calculated to evaluate the total toxic risk of both the acute and chronic toxic effects of SPM heavy metal(loid)s; V and Mn were excluded from the TRI calculations due to the lack of TEL and PEL values. As shown in Figure 6, the TRI values of the 22 sites range from 9.5 (M6) to 32.9 (B1), with a mean value of 17.9, indicating considerable toxic risk for most of the sites (15 < TRI ≤ 20). Additionally, three sites (M7, M16, and B1, TRI > 20) present very high toxic risk, while low toxic risk is observed at M6 (5 < TRI ≤ 10) (Figure 6). In contrast to the EF and I_geo_ values, the mean TRI of individual metal(loid)s follow a decreasing order of As (8.8) > Cd (2.8) > Cr (2.3) > Ni (1.3) > Zn (1.1) > Cu (1.0) > Pb (0.6), with mean contributions of 48.3 ± 10.4%, 15.6 ± 4.3%, 13.0 ± 5.5%, 7.7 ± 3.0%, 6.3 ± 4.5%, 5.8 ± 3.0%, and 3.3 ± 2.1%, respectively, to the TRI, indicating that As accounts for the majority of the overall SPM toxicity. The considerable contributions of As and Cd to the TRI are attributed mainly to their relatively low TEL and high concentration in SPM. This highlights the potential toxicity of SPM in the Zhujiang River, with two metal(loid)s (As and Cd) deserving more concern.

#### 3.4.2. Health Risk Assessment

**Figure 7:**
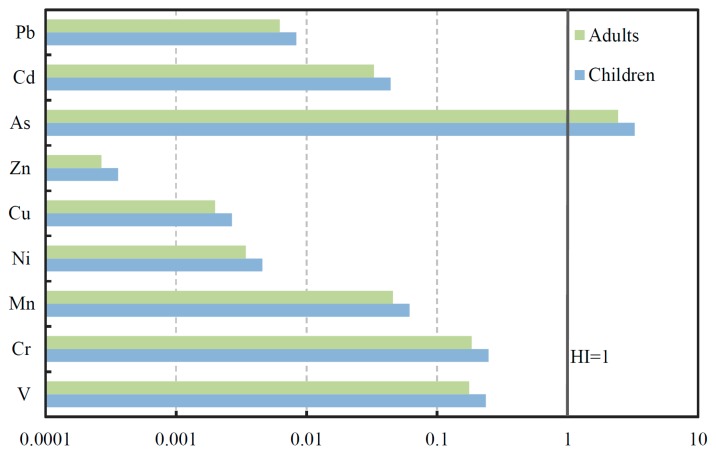
Hazard index (HI) for each metal(loid) of SPM in the Zhujiang River.

To better assess the health risk of human exposure to SPM of the Zhujiang River, the hazard index (HI) for the selected heavy metal(loid)s is calculated based on the reference dose (RfD) of each metal [37,45,60] (Table S2). The mean HI values are shown in Figure 7, and the HI calculated results for each site are summarized in Table S2. It should be noted that mean HI values of As exceed 1 for both children (3.3) and adults (2.4), indicating that non-carcinogenic effects may occur. For both adults and children, the HI for all the metals (except As) are less than 1 (Figure 7, Table S2), indicating that for these metals, little hazard is presented through the only exposure pathway—dermal absorption—in the whole basin area. In general, children have a higher HI value than adults (Figure 7), indicating that children face greater serious health risks due to SPM heavy metals than adults. Additionally, the previous studies concluded that negative health effects may occur for HI values >0.1 in the child cohort [37,61]. Consequently, the V and Cr (with mean HI values of 0.24 and 0.25 for children, Table S2) exposure to the SPM is non-negligible in this study. Considering species-specific toxicity, arsenic (As) mainly afflicts the mucous membrane by directly damaging the capillaries [37,62]; chromium (Cr) can result in asphyxia via reducing oxygen demand of the biochemical process [63]; and vanadium (V) exhibits hepatotoxic, nephrotoxic properties and reproductive system toxicity [64]. Here, we conclude that As is the primary health risk and more attention should also be paid to V and Cr in the Zhujiang River.

#### 3.4.3. Heavy Metal Export Budget Estimation

Based on the concentrations of the heavy metals in SPM and the discharge of the wet season (April to September) at the last site (M18) of the Zhujiang River (River and Sediment Bulletin of China, http://www.mwr. gov.cn/sj/tjgb/zgstbcgb/), river fluxes of each heavy metal in SPM are estimated that range from 38.6 (Cd) to 16,171 (Mn) tons (Table 7). Here, we only calculate the budget of the wet season, and the results may be overestimated due to sampling only once. However, considering that we do not have any samples in the dry season, the overestimated part could approximately equal the export flux of the dry season. Therefore, our results can represent the annual export budget of SPM heavy metal to a certain extent. In combination with the data for dissolved heavy metals [32], the total export budget of each heavy metal was evaluated and decreased in the order of Mn > V > Cr > Ni > Cu > Pb > Cd (Table 7). To eliminate the large uncertainty in evaluation, high-frequency samplings and observations are needed to quantify the annual heavy metal budget, especially in the wet flow season, when the heavy metal concentrations could vary significantly after a storm event.

## 4. Conclusions

In conclusion, this study indicates that systematic analyses of data on nine heavy metal(loid)s in SPM samples of the Zhujiang River using multi-indicators/statistical techniques—including partition coefficient, enrichment factor (EF), geo-accumulation index (I_geo_), toxic risk index, hazard index, correlation analysis and principal component analysis—can provide important support regarding the prevention–control of heavy metal pollution, and health risk control in the whole basin. Our results show that the SPM samples contained high concentrations of several heavy metal(loid)s, including Cr, Mn, Zn, As, and Cd (higher than all soil background values), and the investigated heavy metal(loid)s are powerfully adsorbed by the SPM during water/particle interaction. In particular, the enrichments of As and Cd are noticeable in the SPM, with the consistently high EF and I_geo_ values. Anthropogenic emissions are the main source of the SPM extremely enriched elements (As and Cd), while natural origins are the source responsible for V, Cr, Mn, Ni, Cu, and Pb, and the sources of the remaining heavy metals are controlled by mixed anthropogenic and geologic origins. Moreover, our systematic risk assessment concluded that As could pose potential non-carcinogenic effects on human health and accounted for the majority of the SPM toxicity in the entire catchment. The potential risks of V and Cr with their relatively higher hazard index, is also not negligible. In order to incorporate the possible uncertainty of the single sampling and the variations of geochemical fractions of heavy metal(loid)s in SPM, and to estimate the potential risk clearly, there is a need for further research including high-frequency sampling and heavy metal(loid)s speciation analysis that would help understand the geochemical cycle of heavy metal(loid)s and its environmental effect in the Zhujiang River basin.

## Figures and Tables

**Table 1:** Contamination and toxic risk categories based on enrichment factor (EF), geo-accumulation index (I_geo_) and toxic risk index (TRI).

EF	Enrichment Level	I_geo_	Pollution Intensity	TRI	Toxic Risk
<1	no enrichment	<0	unpolluted	<5	no toxic risk
1–3	minor enrichment	0–1	lightly polluted	5–10	low toxic risk
3–5	moderate enrichment	1–2	moderately polluted	10–15	moderate toxic risk
5–10	moderately severe enrichment	2–3	moderately to heavily polluted	15–20	considerable toxic risk
10–25	severe enrichment	3–4	heavily polluted	>20	very high toxic risk
25–50	very severe enrichment	4–5	heavily to extremely polluted		
>50	extremely severe enrichment	>5	extremely polluted		

**Table 2:** Values and factors used for non-carcinogenic hazard health risk assessment.

Parameter	Physical Meaning	Unit	Children	Adults	Reference
C	Concentration of heavy metal(loid)s in SPM	mg kg^−1^			This study
EF	Exposure frequency	day year^−1^	350	350	[41]
ED	Exposure duration	year	6	30	[41]
SA	Exposed skin area	cm^2^	1800	5000	[37]
AF	Adherence factor	mg cm^−2^ day^−1^	1	1	[37]
ABS	Dermal absorption factor	-	0.03 for As; 0.001 for other metals	0.03 for As; 0.001 for other metals	[37,44]
BW	Average body weight	kg	15	55.9	[37]
AT	Average time	day	365 × ED	365 × ED	[37,44]

**Table 3:** Descriptive statistics of heavy metal(loid)s in suspended particulate matter (SPM) and the SPM concentration of the Zhujiang River (*n* = 22), and the local soil background values of the Zhujiang River basin.

Parameter	V	Cr	Mn	Ni	Cu	Zn	As	Cd	Pb	SPM Concentration
Min	10.9	20.7	152.7	13.1	13.6	49.3	33.5	2.1	8.2	8.0
Max	270.3	221.5	1487.1	62.5	96.4	732.8	317.6	8.9	54.7	944.0
Med	150.5	147.7	1103.6	41.6	36.3	139.1	109.2	3.5	38.6	138.0
AM	143.6	129.1	982.4	39.9	44.1	186.8	116.8	3.8	38.1	177.2
SD	61.5	48.8	379.7	12.0	19.9	138.1	51.6	1.6	11.6	205.5
SGZ	138.8	95.9	794.0	39.1	32.0	99.5	20.0	0.66	35.2	—
SYN	154.9	65.2	626.0	42.5	46.3	89.7	18.4	0.22	40.6	—
SGDGX	97.6	66.3	362.5	20.5	22.4	61.5	14.7	0.16	30.0	—
TEL	—	43.4	—	22.7	31.6	121.0	9.8	1.0	35.8	—
PEL	—	111.0	—	48.6	149.0	459.0	33.0	5.0	128.0	—
K-S test	0.96	0.29	0.55	0.53	0.32	0.14	0.16	0.10	0.65	0.22

Note: Units in mg kg^−1^ for heavy metal(loid)s, mg L^−1^ for SPM concentration; Min, minimum; Max, Maximum; Med, median; AM, arithmetical mean; SD, arithmetical standard deviation; SGZ, soil background values of Guizhou province [36]; SYN, soil background values of Yunnan province [36]; SGDGX, mean soil background values of Guangdong and Guangxi provinces [36]; TEL, threshold effect level [40]; PEL, probable effect level [40]; K–S test, Kolmogorov–Smirnov test; —, no data.

**Table 4:** Comparison of heavy metals in SPM of global rivers (unit in mg kg^−1^).

Rivers	V	Cr	Mn	Ni	Cu	Zn	As	Cd	Pb
Zhujiang River (this study)	143.6	129.1	982.4	39.9	44.1	186.8	116.8	3.8	38.1
World River average	129.0	130.0	1679.0	74.5	75.9	208.0	36.3	1.6	61.1
South American River average	131.0	79.0	700.0	46.0	59.0	184.0	—	—	76.0
North American River average	188.0	115.0	1430.0	50.0	34.0	137.0	—	—	22.0
Asia (Russia) River average	128.0	260.0	5767.0	123.0	145.0	300.0	—	—	35.0
Asia (China) River average	135.0	117.0	970.0	68.0	53.0	145.0	—	—	64.0
Africa River average	116.0	130.0	1478.0	78.0	53.0	130.0	—	—	46.0
Europe River average	85.0	164.0	1884.0	66.0	172.0	346.0	—	—	71.0

Note: The data for global rivers are from Viers et al. [11]; —, no data.

**Table 5:** The partition coefficients (lgK_d_) of heavy metals in the Zhujiang River and global rivers.

River		V	Cr	Mn	Ni	Cu	Cd	Pb
Zhujiang River (this study)	Min	3.6	3.3	4.7	3.7	2.9	4.6	5.4
	Max	5.0	4.5	7.0	4.5	5.3	5.5	6.2
	AM	4.6	4.2	6.3	4.2	4.6	5.0	5.9
Rivers in US		—	5.1	—	4.6	4.7	4.7	5.6
Tigris River		—	6.7	6.6	6.5	6.3	6.3	6.7
Day River		—	5.5	5.0	5.3	5.4	5.7	5.3
Sava River		4.7	4.2	5.9	4.4	3.9	3.0	4.6
Yangtze River		—	4.1	5.0	3.9	4.1	4.2	5.2
Jialingjiang River		—	4.3	5.0	3.8	4.2	4.8	5.1
Beijiang River		—	—	—	—	4.7	4.8	5.2
Upper Zhujiang River		5.4	5.6	6.6	5.3	4.9	5.1	5.7

Note: Min, minimum; Max, maximum; AM, arithmetical mean; —, no data; Rivers in US [53]; Tigris River [6]; Day River [51]; Sava River [50]; Yangtze River and Jialingjiang River [52]; Beijiang River [8]; Upper Zhujiang River [21].

**Table 6:** Varimax rotated component matrix for heavy metal(loid)s of SPM in the Zhujiang River.

Variable	PC 1	PC 2	PC 3	Communalities
V	**0.68**	0.05	−0.31	0.56
Cr	**0.94**	−0.17	−0.16	0.94
Mn	**0.80**	−0.16	0.29	0.75
Ni	**0.83**	−0.45	0.03	0.89
Cu	**0.74**	−0.14	−0.13	0.58
Zn	−0.06	0.04	**0.94**	0.89
As	−0.16	**0.94**	−0.01	0.91
Cd	−0.10	**0.92**	0.05	0.85
Pb	**0.86**	−0.02	0.05	0.75
Eigenvalues	4.01	2.01	1.11	
Variance (%)	44.51	22.36	12.33	
Cumulative (%)	44.51	66.86	79.19	

Note: Extraction method, principal component analysis; Rotation method, Varimax with Kaiser normalization; the “bold” values mean the factor loadings (the correlation coefficients between PC and initial variable) are “strong” or “moderate”.

**Table 7:** Export fluxes of heavy metals (t yr^−1^) and proportions (%) of SPM and the dissolved flux to the total flux in the Zhujiang River.

Parameter	V		Cr		Mn		Ni		Cu		Cd		Pb	
	Flux	%	Flux	%	Flux	%	Flux	%	Flux	%	Flux	%	Flux	%
SPM	3707	83	2585	62	16171	99	709	59	628	78	38.6	77	760.0	99
Dissolved	736	17	1561	38	106	1	498	41	174	22	11.3	23	8.6	1
Total flux	4442		4146		16277		1207		802		50.0		768.6	

## Supplementary Materials

The following are available online at https://www.mdpi.com/1660-4601/16/10/1843/s1, Table S1: Pearson correlation matrix of heavy metal(loid)s of the SPM in the Zhujiang River. Table S2: Hazard index (HI) calculated results for each site, and reference dose for heavy metal(loid)s in the Zhujiang River.
