# Supplementary material for: Heavy Metals in Suspended Particulate Matter of the Zhujiang River, Southwest China: Contents, Sources, and Health Risks
Source: Int J Environ Res Public Health. 2019 May 24;16(10):1843. doi: 10.3390/ijerph16101843 (PMC6572230; doi:10.3390/ijerph16101843)
Supplement: Supplementary file 1 [file ijerph-16-01843-s001.pdf]

Table S1. Pearson correlation matrix of heavy metal(loid)s of the SPM in Zhujiang River.

	V	Cr	Mn	Ni	Cu	Zn	As	Cd	Pb
V	1								
Cr	**0.741****	1							
Mn	0.345	**0.719****	1						
Ni	0.513*	**0.841****	**0.694****	1					
Cu	0.342	**0.679****	0.492*	**0.697****	1				
Zn	-0.154	-0.182	0.089	-0.031	-0.160	1			
As	-0.013	-0.306	-0.310	-0.552**	-0.263	0.060	1		
Cd	-0.159	-0.282	-0.182	-0.457*	-0.136	0.037	**0.780****	1	
Pb	0.430*	**0.783****	**0.696****	**0.692****	**0.546****	-0.079	-0.153	-0.138	1

Note: *, Correlation is significant at the 0.05 level (2-tailed); **, Correlation is remarkably at the 0.01 level (2-tailed).

Table S2. Hazard index (HI) calculated results for each site, and reference dose for heavy metal(loid)s in Zhujiang River.

Site	V	Cr	Mn	Ni	Cu	Zn	As	Cd	Pb
					**Children**				
M1	3.0E-01	2.9E-01	3.6E-02	6.8E-03	3.3E-03	2.5E-04	3.1E+00	4.0E-02	8.7E-03
M2	1.8E-01	2.8E-01	5.4E-02	4.3E-03	4.4E-03	2.5E-04	3.1E+00	4.1E-02	7.5E-03
M3	2.7E-01	3.3E-01	5.8E-02	5.4E-03	4.2E-03	4.1E-04	3.0E+00	4.7E-02	1.2E-02
M4	1.9E-01	2.2E-01	8.4E-02	5.0E-03	3.7E-03	4.2E-04	3.4E+00	3.0E-02	8.3E-03
M5	2.2E-01	2.9E-01	8.7E-02	5.8E-03	4.2E-03	2.4E-04	3.3E+00	4.1E-02	1.0E-02
M6	3.9E-02	4.0E-02	9.6E-03	1.5E-03	1.0E-03	9.5E-05	2.3E+00	3.0E-02	—
M7	1.0E-01	2.4E-01	9.3E-02	4.7E-03	2.8E-03	5.5E-04	4.1E+00	7.4E-02	1.2E-02
M8	1.8E-02	7.6E-02	3.1E-02	1.5E-03	8.2E-04	2.0E-04	2.8E+00	5.2E-02	—
M9	2.9E-01	2.9E-01	7.6E-02	4.7E-03	2.2E-03	2.6E-04	2.9E+00	3.7E-02	8.7E-03
M10	4.4E-01	3.1E-01	7.3E-02	5.2E-03	2.2E-03	2.8E-04	2.6E+00	4.2E-02	7.9E-03
M11	3.2E-01	3.0E-01	6.9E-02	3.9E-03	2.1E-03	2.7E-04	2.7E+00	3.1E-02	1.0E-02
M12	2.5E-01	2.9E-01	6.9E-02	4.5E-03	2.1E-03	2.6E-04	3.0E+00	3.1E-02	7.6E-03
M13	2.9E-01	3.0E-01	7.2E-02	4.8E-03	2.2E-03	3.2E-04	2.8E+00	4.7E-02	7.4E-03
M14	2.2E-01	2.8E-01	5.6E-02	4.8E-03	2.3E-03	6.3E-04	3.1E+00	3.6E-02	4.9E-03
M15	2.6E-01	2.8E-01	7.0E-02	5.4E-03	2.1E-03	3.8E-04	3.8E+00	3.9E-02	6.2E-03
M16	2.2E-01	3.0E-01	8.9E-02	5.0E-03	3.0E-03	4.8E-04	4.0E+00	7.0E-02	1.2E-02
M17	2.5E-01	2.5E-01	7.8E-02	3.9E-03	2.2E-03	2.7E-04	3.5E+00	4.5E-02	1.8E-03
M18	3.3E-01	2.6E-01	5.4E-02	4.4E-03	2.0E-03	2.6E-04	3.7E+00	2.4E-02	8.9E-03
B1	2.8E-01	1.2E-01	1.2E-02	—	1.9E-03	2.2E-04	8.9E+00	1.0E-01	—
B2	3.8E-01	4.2E-01	8.9E-02	7.2E-03	5.8E-03	2.7E-04	9.4E-01	2.5E-02	8.5E-03
B3	1.9E-01	1.8E-01	5.0E-02	4.8E-03	3.0E-03	1.7E-04	1.7E+00	4.6E-02	—
B4	1.5E-01	8.1E-02	4.3E-02	2.8E-03	1.2E-03	1.4E-03	3.4E+00	4.0E-02	—
Min	1.8E-02	4.0E-02	9.6E-03	1.5E-03	8.2E-04	9.5E-05	9.4E-01	2.4E-02	1.8E-03
Max	4.4E-01	4.2E-01	9.3E-02	7.2E-03	5.8E-03	1.4E-03	8.9E+00	1.0E-01	1.2E-02
AM	**2.4E-01**	**2.5E-01**	6.1E-02	4.6E-03	2.7E-03	3.6E-04	**3.3E+00**	4.4E-02	8.3E-03
					**Adults**				
M1	2.3E-01	2.2E-01	2.7E-02	5.1E-03	2.4E-03	1.9E-04	2.3E+00	3.0E-02	6.5E-03
M2	1.3E-01	2.1E-01	4.0E-02	3.2E-03	3.3E-03	1.8E-04	2.3E+00	3.0E-02	5.6E-03
M3	2.0E-01	2.5E-01	4.3E-02	4.0E-03	3.1E-03	3.0E-04	2.2E+00	3.5E-02	8.6E-03
M4	1.4E-01	1.7E-01	6.3E-02	3.7E-03	2.7E-03	3.1E-04	2.6E+00	2.3E-02	6.2E-03
M5	1.6E-01	2.1E-01	6.5E-02	4.3E-03	3.1E-03	1.8E-04	2.4E+00	3.1E-02	7.5E-03
M6	2.9E-02	3.0E-02	7.1E-03	1.2E-03	7.7E-04	7.0E-05	1.7E+00	2.2E-02	—
M7	7.8E-02	1.8E-01	6.9E-02	3.5E-03	2.1E-03	4.1E-04	3.1E+00	5.5E-02	8.8E-03
M8	1.3E-02	5.7E-02	2.3E-02	1.1E-03	6.1E-04	1.5E-04	2.1E+00	3.9E-02	—
M9	2.1E-01	2.2E-01	5.6E-02	3.5E-03	1.6E-03	2.0E-04	2.2E+00	2.8E-02	6.5E-03
M10	3.3E-01	2.3E-01	5.4E-02	3.9E-03	1.6E-03	2.1E-04	2.0E+00	3.2E-02	5.9E-03
M11	2.4E-01	2.2E-01	5.1E-02	2.9E-03	1.6E-03	2.0E-04	2.0E+00	2.3E-02	7.6E-03
M12	1.8E-01	2.2E-01	5.1E-02	3.4E-03	1.6E-03	2.0E-04	2.2E+00	2.3E-02	5.7E-03

Site	V	Cr	Mn	Ni	Cu	Zn	As	Cd	Pb
M13	2.2E-01	2.2E-01	5.3E-02	3.6E-03	1.6E-03	2.3E-04	2.1E+00	3.5E-02	5.5E-03
M14	1.7E-01	2.1E-01	4.2E-02	3.6E-03	1.7E-03	4.7E-04	2.3E+00	2.7E-02	3.6E-03
M15	2.0E-01	2.1E-01	5.2E-02	4.0E-03	1.6E-03	2.8E-04	2.8E+00	2.9E-02	4.6E-03
M16	1.7E-01	2.2E-01	6.6E-02	3.7E-03	2.2E-03	3.6E-04	3.0E+00	5.2E-02	8.9E-03
M17	1.8E-01	1.9E-01	5.8E-02	2.9E-03	1.7E-03	2.0E-04	2.6E+00	3.3E-02	1.3E-03
M18	2.4E-01	2.0E-01	4.0E-02	3.2E-03	1.5E-03	1.9E-04	2.8E+00	1.8E-02	6.6E-03
B1	2.1E-01	8.9E-02	8.6E-03	—	1.4E-03	1.6E-04	6.6E+00	7.6E-02	—
B2	2.9E-01	3.2E-01	6.6E-02	5.4E-03	4.4E-03	2.0E-04	7.0E-01	1.9E-02	6.3E-03
B3	1.4E-01	1.3E-01	3.8E-02	3.6E-03	2.2E-03	1.3E-04	1.2E+00	3.4E-02	—
B4	1.1E-01	6.1E-02	3.2E-02	2.1E-03	8.9E-04	1.0E-03	2.5E+00	3.0E-02	—
Min	1.3E-02	3.0E-02	7.1E-03	1.1E-03	6.1E-04	7.0E-05	7.0E-01	1.8E-02	1.3E-03
Max	3.3E-01	3.2E-01	6.9E-02	5.4E-03	4.4E-03	1.0E-03	6.6E+00	7.6E-02	8.9E-03
AM	1.8E-01	1.8E-01	4.6E-02	3.4E-03	2.0E-03	2.7E-04	**2.4E+00**	3.3E-02	6.2E-03
**RfD**	7.0E-05	6.0E-05	1.8E-03	1.0E-03	1.9E-03	6.0E-02	1.2E-04	1.0E-05	5.3E-04

Note: Units in mg kg^{-1} day^{-1} for RfD; Min, minimum; Max, Maximum; AM, arithmetical mean; RfD, reference dose [1,2]; "—" means HI value is not calculated due to the metal contents in the corresponding SPM sample are below detectable limit.

References

1. Wu, T.; Bi, X.; Li, Z.; Sun, G.; Feng, X.; Shang, L.; Zhang, H.; He, T.; Chen, J. Contaminations, Sources, and Health Risks of Trace Metal(loid)s in Street Dust of a Small City Impacted by Artisanal Zn Smelting Activities. International Journal of Environmental Research and Public Health 2017, 14, 961, doi:10.3390/ijerph14090961.
2. Wan, D.J.; Zhan, C.L.; Yang, G.L.; Liu, X.Q.; Yang, J.S. Preliminary Assessment of Health Risks of Potentially Toxic Elements in Settled Dust over Beijing Urban Area. International Journal of Environmental Research and Public Health 2016, 13, doi:10.3390/ijerph13050491.
